# Integrated computer-aided drug design and biophysical simulation approaches to determine natural anti-bacterial compounds for *Acinetobacter baumannii*

**DOI:** 10.1038/s41598-022-10364-z

**Published:** 2022-04-21

**Authors:** Raed A. H. Almihyawi, Ziad Tareq Naman, Halah M. H. Al-Hasani, Ziyad Tariq Muhseen, Sitong Zhang, Guang Chen

**Affiliations:** 1 College of Life Sciences, Jilin Agricultural University, Jilin, China; 2 Department of Quality Control, Baghdad Water Authority, Mayoralty of Baghdad, Baghdad, Iraq; 3 Department of Medical Laboratory Techniques, Al Mamoon University College, Baghdad, Iraq; 4 Department of Biotechnology, College of Science, University of Diyala, Baqubah, Iraq; 5 Department of Biomedical Engineering, College of Engineering and Applied Sciences, Nanjing University, Nanjing, 210093 China; 6 Key Laboratory of Straw Biology and Utilization, Ministry of Education, Jilin, China

**Keywords:** Biophysics, Computational biology and bioinformatics, Drug discovery

## Abstract

*Acinetobacter baumannii* is a nosocomial bacterial pathogen and is responsible for a wide range of diseases including pneumonia, necrotizing fasciitis, meningitis, and sepsis. The enzyme 5-enolpyruvylshikimate-3-phosphate (EPSP) synthase (encoded by aroA gene) in ESKAPE pathogens catalyzes the sixth step of shikimate pathway. The shikimate pathway is an attractive drug targets pathway as it is present in bacteria but absent in humans. As EPSP is essential for the *A. baumannii* growth and needed during the infection process, therefore it was used as a drug target herein for high-throughput screening of a comprehensive marine natural products database (CMNPD). The objective was to identify natural molecules that fit best at the substrate binding pocket of the enzyme and interact with functionally critical residues. Comparative assessment of the docking scores allowed selection of three compounds namely CMNPD31561, CMNPD28986, and CMNPD28985 as best binding molecules. The molecules established a balanced network of hydrophobic and hydrophilic interactions, and the binding pose remained in equilibrium throughout the length of molecular simulation time. Radial distribution function (RDF) analysis projected key residues from enzyme active pocket which actively engaged the inhibitors. Further validation is performed through binding free energies estimation that affirms very low delta energy of <−22 kcal/mol in MM-GBSA method and <−12 kcal/mol in MM-PBSA method. Lastly, the most important active site residues were mutated and their ligand binding potential was re-investigated. The molecules also possess good druglike properties and better pharmacokinetics. Together, these findings suggest the potential biological potency of the leads and thus can be used by experimentalists in vivo and in vitro studies.

## Introduction

Antimicrobial resistance (AMR) by bacterial pathogens has made infection management less effective, increased health care costs, need more intense care, lengthen hospital stay, and enhanced mortality rate^1,2^. Among the bacterial pathogens, *Acinetobacter baumannii* is considered as a superbug and is listed top on the global priority list of bacterial pathogens because of its good potential of resisting almost every antibiotic^3,4^. *A. baumannii* is a gram-negative, non-motile, aerobic bacillus that often causes respiratory, wound, and blood stream infections, particularly in immune-compromised populations^5^. Multi-drug resistant *A. baumannii* (MDR-AB) outbreaks are usually common and have been reported in countries such as Brazil, China, the United Kingdom, the United States, India, Spain, Germany, Iran, Turkey, and Iraq^6,7^. The pathogen colonizes 75% of hospitalized individuals and 42.5% of healthy individuals^8^. More worrisome are reports describing the emergence of Carbapenems such as imipenem and meropenem resistant phenotypes of *A. baumannii* across the globe^9^. Such Carbapenem resistant *A. baumannii* (CR-AB) are responsible for 26 to 76% of mortality of infants in intensive care units (ICU)^10^. Severe MDR-AB and CR-AB are treated with last resort Colistin antibiotic however, its use is limited as it causes neurotoxicity and nephrotoxicity^11^. Tigecycline is the first US FDA approved effective antibiotic against CR-AB^12^. Tigecycline is mostly used as a treatment for severe problems such as skin and intra-abdominal infections and community acquired pneumonia etc^13^. Due to the bacteriostatic activity of Tigecycline against MDR-AB, it is usually prescribed as a final drug and inhibits bacterial protein synthesis by interfering with the attachment of aminoacyl-tRNA at the A site of the 30S ribosome^14^. Alarmingly, recent times have witnessed an increased emergence of Tigecycline resistance in *A. baumannii* (TRAB) as well, therefore, demanding the identification of newer drug targets against which new drug molecules could be designed^15,16^.

As traditional drug discovery process is time consuming and costly, bioinformatics approaches are gaining great attention to identify novel drug targets that are specific and selective against bacterial pathogens^17–19^. As an example, using bioinformatics techniques seven metabolic pathway enzymes and 15 non-homologous membrane proteins were discovered as promising antibacterial targets against *Staphylococcus aureus*^20^. Existing targets are also useful to be explored for unveiling new classes of drug molecules^21^. This can be explained by oxadiazoles drug identification against penicillin-binding protein 2a of Methicillin-resistant *S. aureus* (MRSA)^22^. Telithromycin, a third-generation ketolide antibiotic, was identified by Andrade and coworkers as a specific antibacterial molecule against bacterial resistant strains^23^. In short, lead compounds can be screened by combine applications of computer aided drug designing and using knowledge of medicinal chemistry for accumulating structure activity information to facilitate the development of next-generation of antibiotics^17,19,24^.

In this present in silico study, the aim is to identify molecules from natural marine organisms as selective inhibitors *A. baumannii* 5-enolpyruvylshikimate-3-phosphate (EPSP) synthase of shikimate pathway^25^. The shikimate pathway is an attractive drug target pathway as it is present in bacteria, absent in humans, and essential for bacterial growth and survival^26^. Employing various servers and databases, several drug molecules are proposed. Site directed based virtual screening was performed to shortlist best docked molecules^27,28^, which were analyzed further in biophysical analysis of molecular dynamics (MD) simulation^29,30^ and comprehensive binding free energies calculations^31^ via molecular mechanics energies combined with the Poisson–Boltzmann or generalized Born and surface area continuum solvation (MM-PBSA and MM-GBSA) methods^27,32,33^. The findings may be helpful for future biological activity optimization and lead discovery.

## Materials and methods

**Figure 1:**
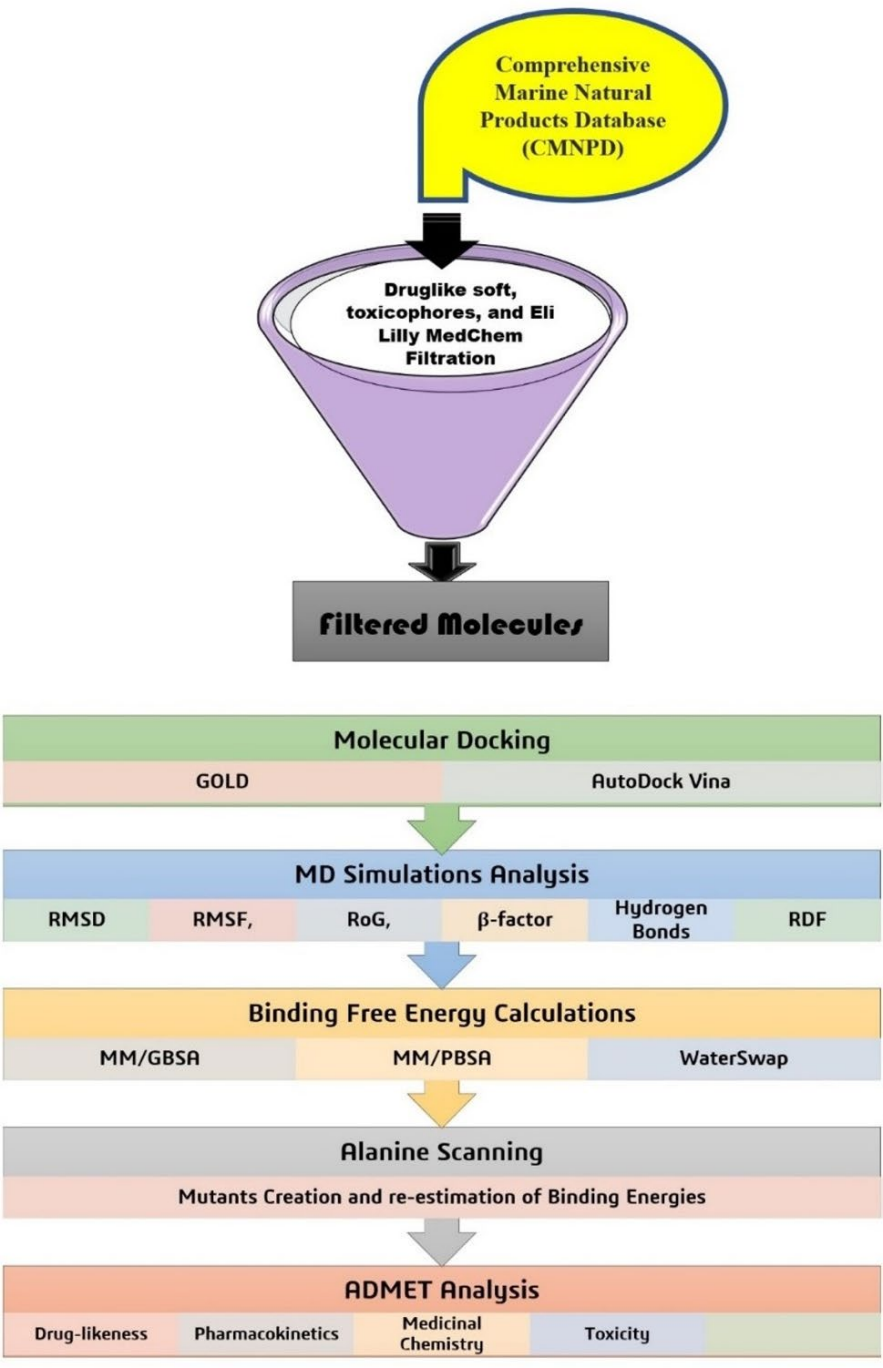
Methodology flowchart to achieved docking, dynamics, and ADMET analysis of virtual screened compounds.

The flow chart as shown in Fig. 1 summarizes the study methodology. Potential drug molecules were filtered and subsequently used in different applications of computer aided drug designing (CADD)^17,34,35^ to investigate their potency to block biological functionality of EPSP synthase enzyme.

### Retrieval EPSP synthase crystal structure and preparation for docking studies

Crystal structure of EPSP synthase enzyme was retrieved from Protein Data Bank (PDB)^36^. The structure is determined at a resolution of 2.37 Å, R-value of 0.186, and submitted under the PDB ID of 5BUF^25^. To prepare the enzyme structure for molecular docking, extra chain, water molecules, and co-crystallized ligands were removed in UCSF Chimera version 1.15^37^. The structure energy was then minimized through 1000 steepest descent and conjugate gradient algorithms keeping step size value to 0.02 Å. AMBER ff14SB^38^ was utilized as a force field to parameterize enzyme backbone and side chains residues. To assess the general quality of the minimized structure, PDBSum Ramachandran plot^39^ was generated and compared with the unminimized. The superimposed energy minimized EPSP enzyme over experimental EPSP enzyme is provided as Fig. S1.

### CMNPD preparation

To identify natural inhibitors against EPSP synthase enzyme, CMNPD database (https://www.cmnpd.org/) ^40^ was considered. This manually curated database is freely accessible and considered a promising source of valuable novel leads collected from marine organisms. The CMNPD database comprises approximately 47,000 molecules of bacterial, fungal, and algae origin. The database was retrieved as .sdf and then consequently filtered in an online FAFDrugs4 server^41^ where druglike soft, toxicophores, and Eli Lilly MedChem rules were applied in step-wise fashion to eliminate non drug-like, toxic^42^ and PAINS compounds^43^, respectively. Afterward, the filtered compounds were imported to PyRx 0.8^44^, energy minimized, and converted to .pdbqt making the compounds ready to be used in molecular docking studies.

### Site directed virtual screening (SDVS)

SDVS of the filtered library against EPSP synthase enzyme was performed using two popular docking softwares; Genetic Optimization for Ligand Docking (GOLD) 5.2^45^ and PyRx AutoDock Vina^44,46^ to cross validate the docking prediction and select the best binding molecules based on consensus docking scores. In both docking methods, rigid docking approach was utilized. The binding spot was selected by centering grid box at Ser238 OG atom (X-axis: −0.544 Å, Y-axis: 28.321 Å, and Z-axis: −12.451 Å) with 15 Å dimensions. For each molecule, 100 iterations were produced and assigned with Vina docking score in AutoDock Vina and GOLD fitness score in GOLD. The complexes were compared and frequent three hits were selected based on highest GOLD fitness score and lowest Vina docking score in kcal/mol. The protein with dock box is provided as Fig. S2. The docking protocol was validated by docking N-[phosphomethyl]glycine (glyphosate) inhibitor to the enzyme active pocket and docking score and binding energy value were calculated. The best docked molecules complex were retrieved in PDB and visualized in UCSF Chimera 1.15^37^ and Discovery Studio (DS) Visualizer v21.1.0.20298^47^.

### EPSP-inhibitors dynamics analysis

Top hit docked complexes were subjected to MD simulations for investigating intermolecular affinity in dynamics on a time scale of 100 ns. The AMBER20^48^ was utilized for MD simulations. The Ff14SB force field^38,49^ was employed to prepare parameters of EPSP while AMBER General Force Field (GAFF)^50^ was used for compounds processing. The complexes were placed into TIP3P water box (12 Å in size) which provided a neutral environment as it contains an appropriate number of counterions. The TIP3P water model offers better performance in calculating specific heats compared to other water models^51^. Hydrogen atoms, solvation box, carbon alpha atoms and all non-heavy atoms of the complexes were energy minimized for 1000 steps. Each system temperature was increased gradually to 310 K for 20 ps in Langevin dynamics^52^. This was followed by equilibration for 100 ps and production run of 100 ns. AMBER CPPTRAJ module^53^ was employed to examine complexes stability by plotting different structural deviations analysis versus time. Visual molecular dynamics (VMD) software^54^ was used to plot hydrogen bonds formed in each frame of MD simulation trajectories between enzyme and inhibitor.

### Pair correlation function -g (r)

Pair correlation function (PCF), a radial distribution function, is a highly significant parameter in MD simulations to compute the average interaction density distribution of ligand atom(s) around specific receptor atom(s)^55^. PCF plots were generated for hydrogen bonds between EPSP active pocket residues key to the binding of inhibitors. The PCF analysis was executed using CPPTRAJ and can be presented as,

$$g(r) = \frac{\rho {\mathrm{ij}}({\mathrm{r}})}{< \rho {\mathrm{j}} > }=\frac{nij (r)}{ < \rho {\mathrm{j}} > 4\uppi {\mathrm{r}}^{2}\delta {\mathrm{r}}}$$

where, ρij is the density of a given receptor atom at distance “r” of the ligand atom. The g (r) functions as a ratio between observed interaction density “ρij” at distance “r” and density of solvent bulk atom “ρj”. This ratio is equivalent to ratio between nij(r) and < ρj > 4π ${\mathrm{r}}^{2}$ δr. Nij represents the number of bin atoms in spherical volume fragment depending on their width δr. The *4πr2δr* is the spherical shell volume having thickness “δr” and at distance “r” from a reference solute atom.

### MM-PB/GBSA studies

MMPBSA.py package^56^ of AMBER20 was employed to estimate binding free energies of the systems^27,32^. The main purpose of this analysis was to find out the difference of free energy between two states of the complex i.e. solvated and gas phase using the below equation,

$$\Delta G\;{\text{binding}}\;{\text{free}}\;{\text{energy}} = \Delta G\;{\text{binding}},\;{\text{vaccum}} + \Delta G\;{\text{solvation}},\;{\text{complex}} - \left( {\Delta G\;{\text{solvation}},\;{\text{ligand}} + \Delta G\;{\text{solvation}},\;{\text{receptor}}} \right)$$

From complete simulation trajectories, 100 frames were used as input in both MM/PBSA and MM/GBSA. The selection of 100 frames was done using an input parameter file of AMBER20 MM-GB/PBSA which allowed considering 100 frames from simulation trajectories picked at an equal time interval. The dielectric constant used in MMPBSA.py was 1. Calculation of entropy energy contribution to each complex binding free energy was done using a bash script given by Duan et al.^57^. The total binding energy of MM-GBSA was further decomposed into the net energy contribution of each enzyme residue that is involved in inhibitor interactions.

### Cross-validation of binding energy by WaterSwap

A more sophisticated approach called WaterSwap^58,59^ was run further to cross-validate MM-PB/GBSA binding free energies. WaterSwap method uses three algorithms Thermodynamic Integration (TI), Free Energy perturbation (FEP), and Bennett’s acceptance ratio (BAR) methods to compute system binding energy for default 1000 iterations. The difference in average binding energy value of each of the above methods for the systems is < 1 kcal/mol which demonstrates the systems well converged.

### Alanine scanning analysis

Specific residues involved in consistent interactions and stability of complexes were selected for alanine scanning analysis, which was performed using AMBER20^60^. Functional significant residues were targeted based on docking interactions and residue-wise decomposition of MM-GBSA binding free energy. The residues were manually replaced with ALA coordinates and the structures were loaded into LEAP module of AMBER^61^. The initial parameter and coordinate files were generated for a short 20 ns of simulation and subsequent analysis of MM-GBSA was performed. The goal was to look for fluctuations in binding free energies as a result of mutation of the mentioned residues. Details of the alanine scanning methods used in this study can be found in a study carried out by Asma et al.^61^.

### Pharmacokinetics studies

Physicochemical properties, medicinal chemistry, druglikeness, and Absorption, Distribution, Metabolism, Excretion and Toxicity (ADMET) analysis of the shortlisted compounds were performed through online servers i.e. pkCSM^62^ and SwissADME^63^.

## Results and discussion

### Selecting a high quality protein structure

**Figure 2:**
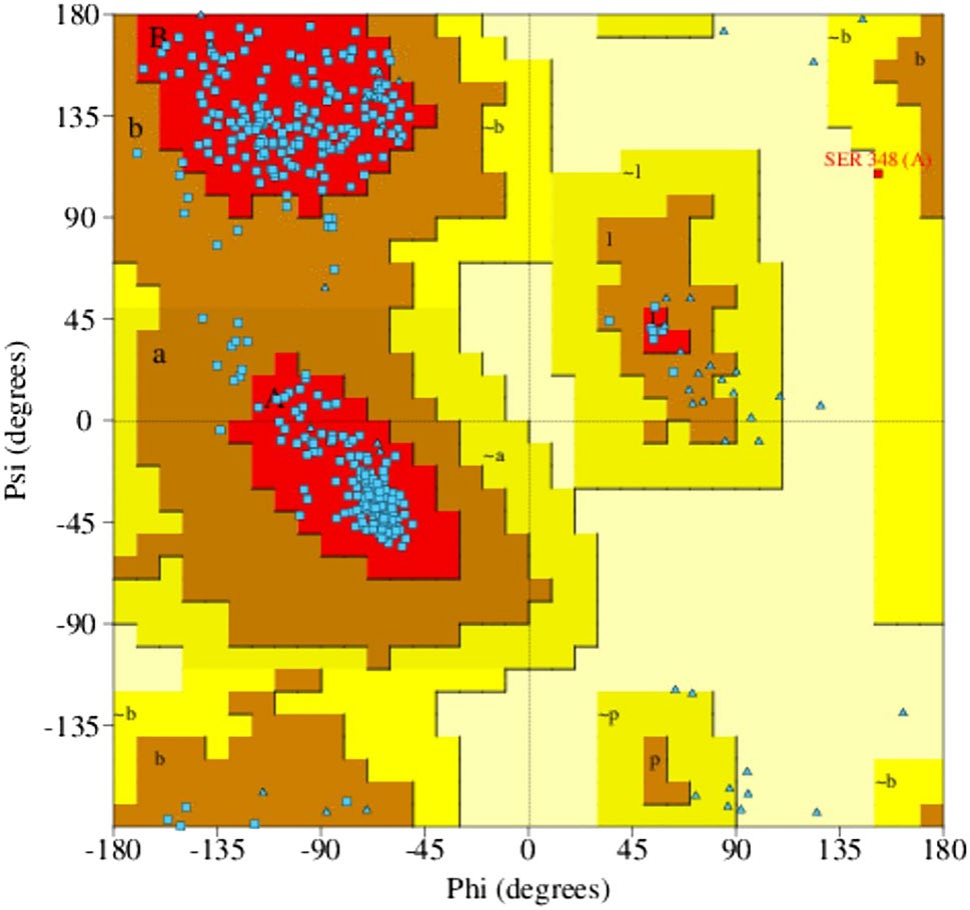
Energy minimized EPSP Ramachandran plot. Details about the coloring of the plot can be interpreted from PDBSum generate server^39^.

To EPSP enzyme structure steric clashes were removed by energy minimization process. These steric clashes are high-energy conformations that can trigger physical perturbation and complex instability during simulation^64,65^. However, such minimization may introduce bad contacts in the structure, affecting the overall enzyme structure. As a result, before and after minimization evaluation of the enzyme is critical in determining energy optimized structure for consequent docking and simulation to make the most accurate predictions possible. Pre-minimized of the EPSP Ramachandran plot had 87.21%residues in the favored region, while the energy minimized enzyme Ramachandran plot had 90.7% residues in the favored region. Furthermore, 1% of residues and 0% of residues were plotted in disallowed regions of the Ramachandran plot of pre-minimized and minimized structures, respectively. The energy minimized EPSP Ramachandran plot is presented in Fig. 2.

### Unveiling EPSP synthase inhibitory molecules

**Figure 3:**
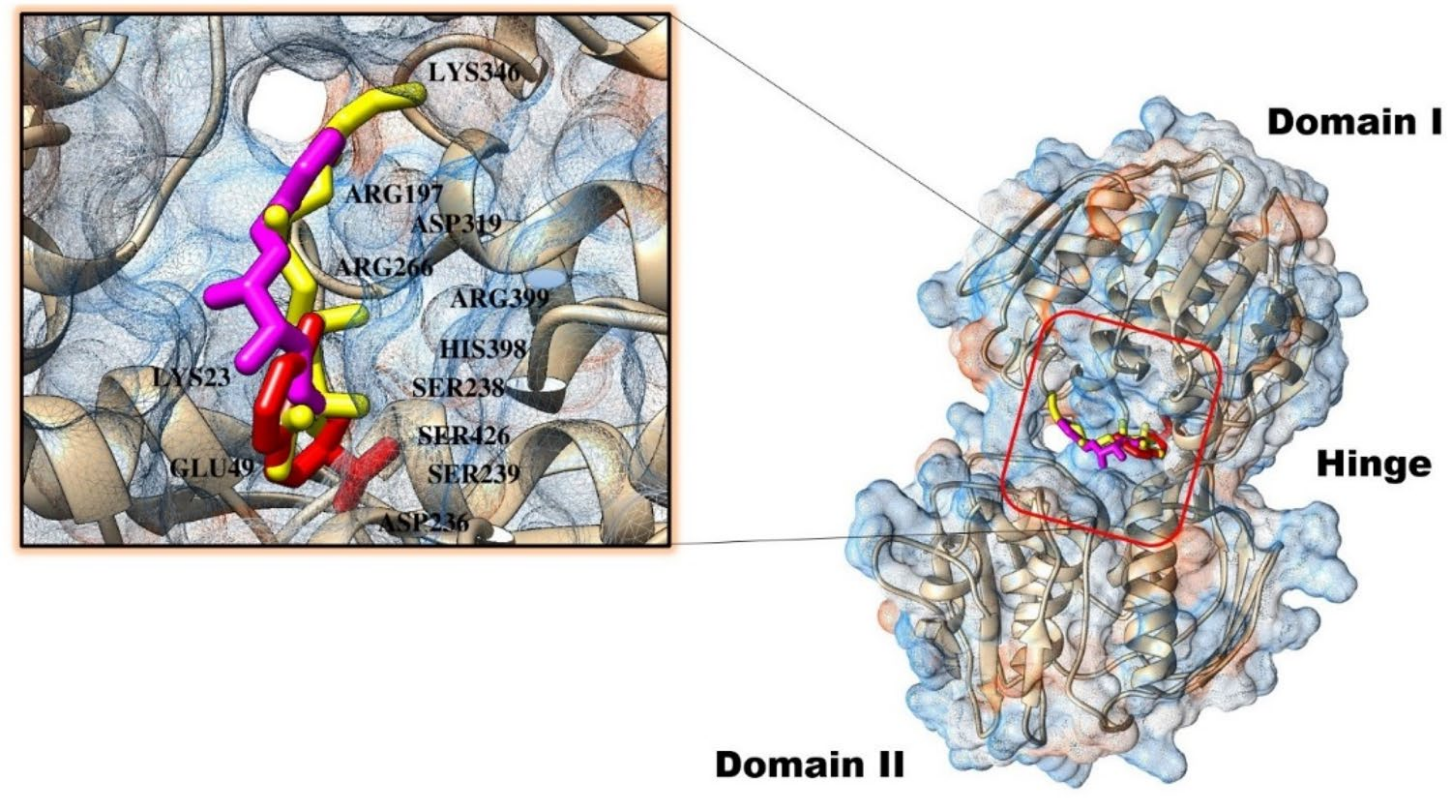
Intermolecular docked pose of the complexes. The enzyme is mesh surface and presented as cartoon. The compounds are colored in sticks. Top-1 (yellow), Top-2 (red) and Top-3 (magenta).

SDVS yielded three potential and promising inhibitory compounds: Top-1 (CMNPD31561), Top-2 (CMNPD28986), and Top-3 (CMNPD28985) depicting Vina docking score of −8.1 kcal/mol, −7.9 kcal/mol, and −7.7 kcal/mol, respectively. The GOLD fitness score of the compounds is 72.54 for Top-1, 70.87 for Top-2, and 70.12 for Top-3. In contrast, the N-[phosphomethyl]glycine (glyphosate) inhibitor binding energy and GOLD fitness score were −6.3 kcal/mol and 69.23, respectively. Selection of the compounds was based on interactions at the binding site of EPSP and plausible suitable binding pose. Balance interactions of both hydrophilic and hydrophobic nature were witnessed between the compound’s chemical moieties and several amino acid residues of the enzyme active pocket. All the three compounds were revealed to be docked at the hinge interface of enzyme and formed close distance contacts with residues Lys23, Glu49, Arg197, Ser238, Ser239, Arg266, Asp319, Lys346, His398, Arg399, and Ser426 (Fig. 3).

**Figure 4:**
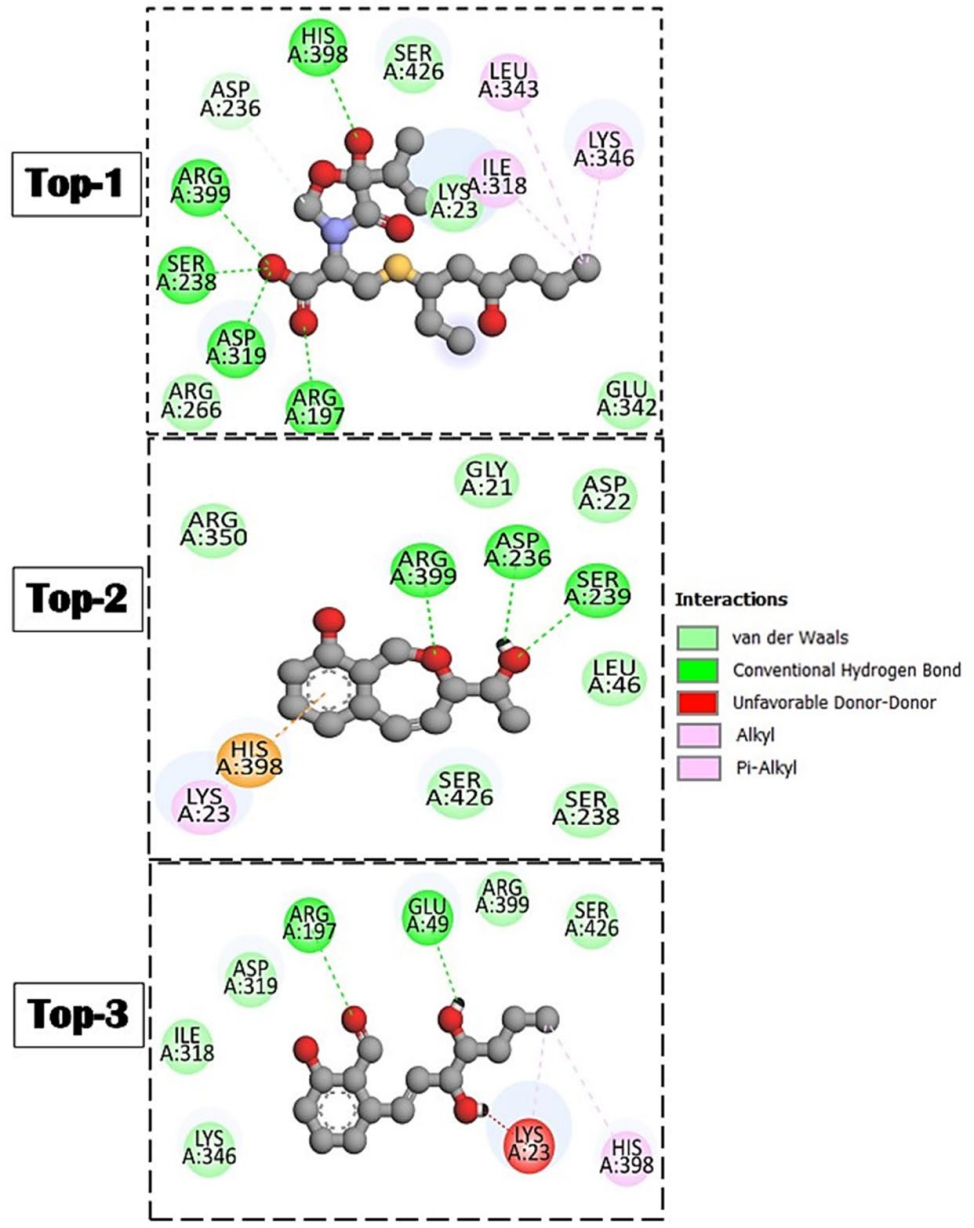
Chemical interactions produced by compounds at the docked pocket of EPSP enzyme. As can be seen, several van der Waals and hydrogen bond interactions were observed as key to stable binding of the compound to the enzyme.

Top-1 upon binding produced several key hydrogen bonding with Arg197, Ser238, His398, and Arg399. Top-2 on the other hand, shared the same binding residue Arg399 as visualized in Top-1 but additionally formed hydrogen bonding with Asp236 and Ser239. Top-3 interacts with two residues (Glu49 and Arg197) of the active pocket. Besides these good number of hydrogen bonding, each compound interacts hydrophobically with many residues of the enzyme active site which significantly contribute to the overall compounds stable binding mode at the enzyme active pocket. The chemical interactions of the compounds with enzyme active pocket are given in Fig. 4.

### MD simulation analysis

**Figure 5:**
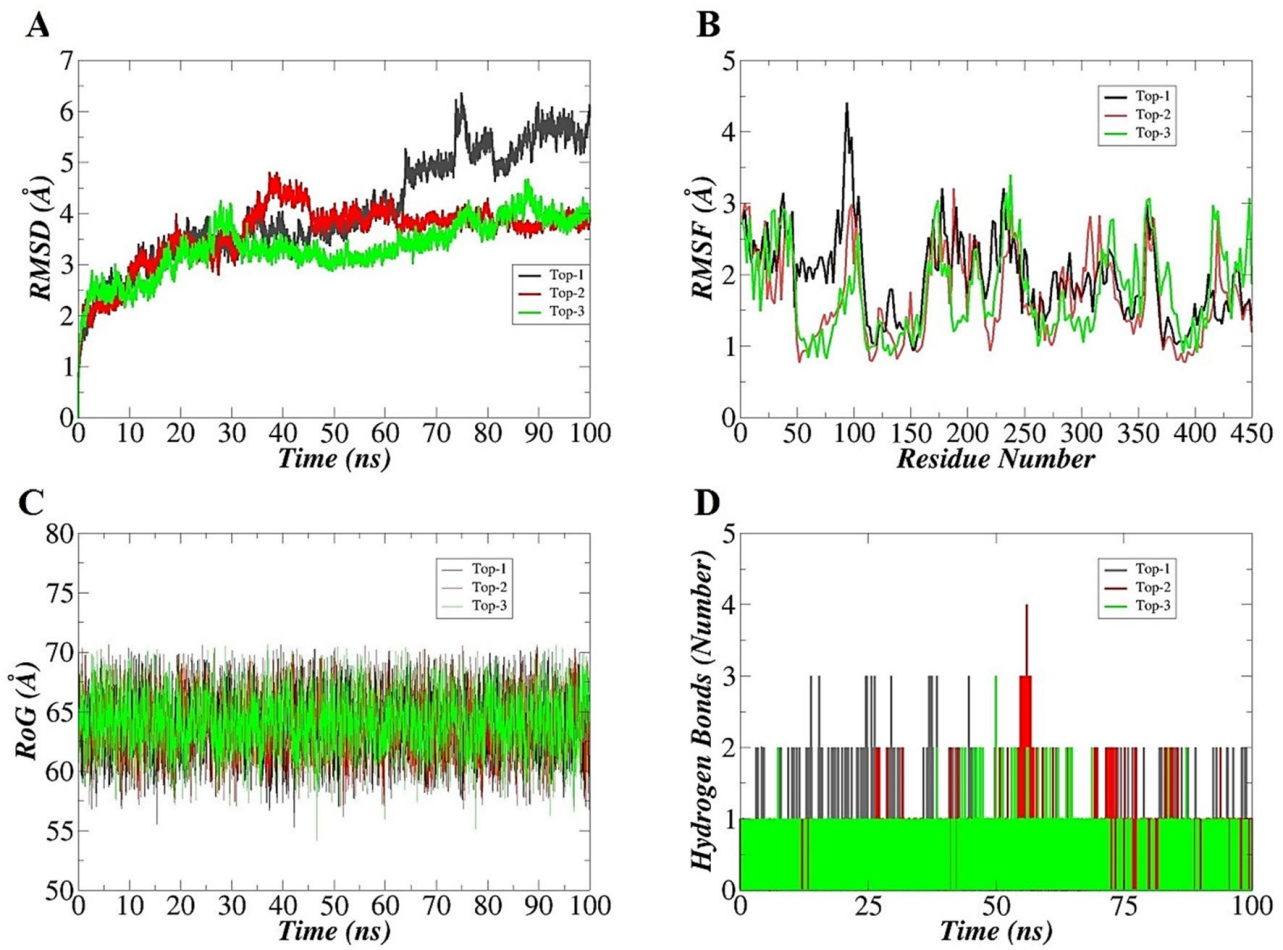
Investigating docked conformational stability of enzyme-compounds complexes in dynamics via different statistical parameters. (**A**) RMSD, (**B**) RMSF, (**C**) RoG, and (**D**) hydrogen bonding.

MD simulations^66^ experiment was performed to further evaluate the conformational strength of hit molecules with EPSP for 100 ns. The conformational stability of complexes was investigated first by root mean square deviations (RMSD)^67^ that measures all carbon alpha atoms deviations considering the docked conformation of the complexes as a reference (Fig. 5A). Compared to Top-1, Top-2 and Top-3 are predicted to form considerably stable complexes besides small structural fluctuations. Both these systems are well converged, and the inhibitor molecules are well stable inside the active pocket. RMSD of both systems touches ~ 4 Å. For Top-3, a minor sharp RMSD spike was noticed near 30 ns touching RMSD of 4 Å, then followed a lower RMSD pattern of 3 Å till 75 ns. Another structure variation was captured at 88 ns with RMSD > 4 Å and towards the end, the RMSD acquired equilibrium. Frames inspection at the fluctuating RMSD time determined no effect on the compound binding and the binding mode of the compound is firmed as predicted originally by docking. These variations correspond to the structural adjustments made by the enzyme in particular by the movement of domain I and domain II and trying to hold the docked molecule by more strength. Top-2 compound is seen with uniform RMSD throughout simulation time with only small window structural RMSD upsurge of ~ 4.7 Å. Again, this structure movement of the enzyme is not affecting compound binding conformation. Top-1 compound RMSD is constant till 60 ns, followed by fluctuations till 80 ns (mediated by enzyme flexible loops) and system stabilization towards the simulation end. For Top-1, the mean RMSD is 4.06 Å (standard deviation, 0.60), Top-2 mean RMSD is 3.60 Å (standard deviation, 0.60), and Top-3 mean RMSD is 3.32 Å (standard deviation, 0.64). The mean RMSD is an easy interpretation of overall stable or unstable nature and herein, as the mean values are in acceptable range, the systems are stable in terms intermolecular conformation and binding interactions. In case of Top-1, the mean RMSD for domain I and II is 2.8 Å and 2.6 Å, respectively while for Top-2, RMSD is 2.0 Å (domain I) and 1.9 Å (domain II) and Top-3, the RMSD is 1.7 Å (domain I) and 1.67 Å (domain II). Next, root mean square fluctuation (RMSF)^68^ was determined for the systems that describe fluctuations of protein residues from the original position during simulation. In this analysis, residues of the protein active key in binding inhibitor were also elucidated. As can be analyzed from Fig. 5B, residues of the enzyme are subject to continuous dynamics however, the fluctuations are within acceptable range (~ 3 Å) and contributing to good stable enzyme conformation in the presence of compounds. These fluctuations are the outcome as discussed above due to the larger size of the enzyme and dynamically more active domains^69,70^. The continuous motions of the enzyme domains are natural for enzyme functionality but are not affecting compounds binding and overall chemical interactions network^31^. The average RMSF of Top-1, Top-2, and Top-3 is 1.86 Å, 1.62 Å and1.76 Å, respectively. This reflects on the good overall stability of the enzyme residues in the presence of compounds during simulation time. Systems RMSF correlated with their respective RMSD plots and complement each other in deciphering complex dynamics. Radius of gyration (RoG)^71^ was performed next to explain compactness and relaxation of the enzyme structure during simulation (Fig. 5C). Increased variation of RoG plot indicates loss of compactness in the structure and vice versa. Average RoG of the systems observed is 63.68 Å with a standard deviation of 2.39 (Top-1), 63.72 Å with a standard deviation of 2.41 (Top-2), and 64.02 Å with a standard deviation value of 2.34 (Top-3). RoG also investigated some minor structural variations that are because of the enzyme loops, which are naturally flexible however, these alterations do not alter stability to the compounds at the docked pocket. Lastly, the number of hydrogen bonds the compounds made to the enzyme active pocket residues were evaluated^72^ (Fig. 5D). All the compounds formed a good number of hydrogen bonds with enzyme active pocket residues that are consistent and of close distance suggesting higher intermolecular affinity of the complexes.

**Figure 6:**
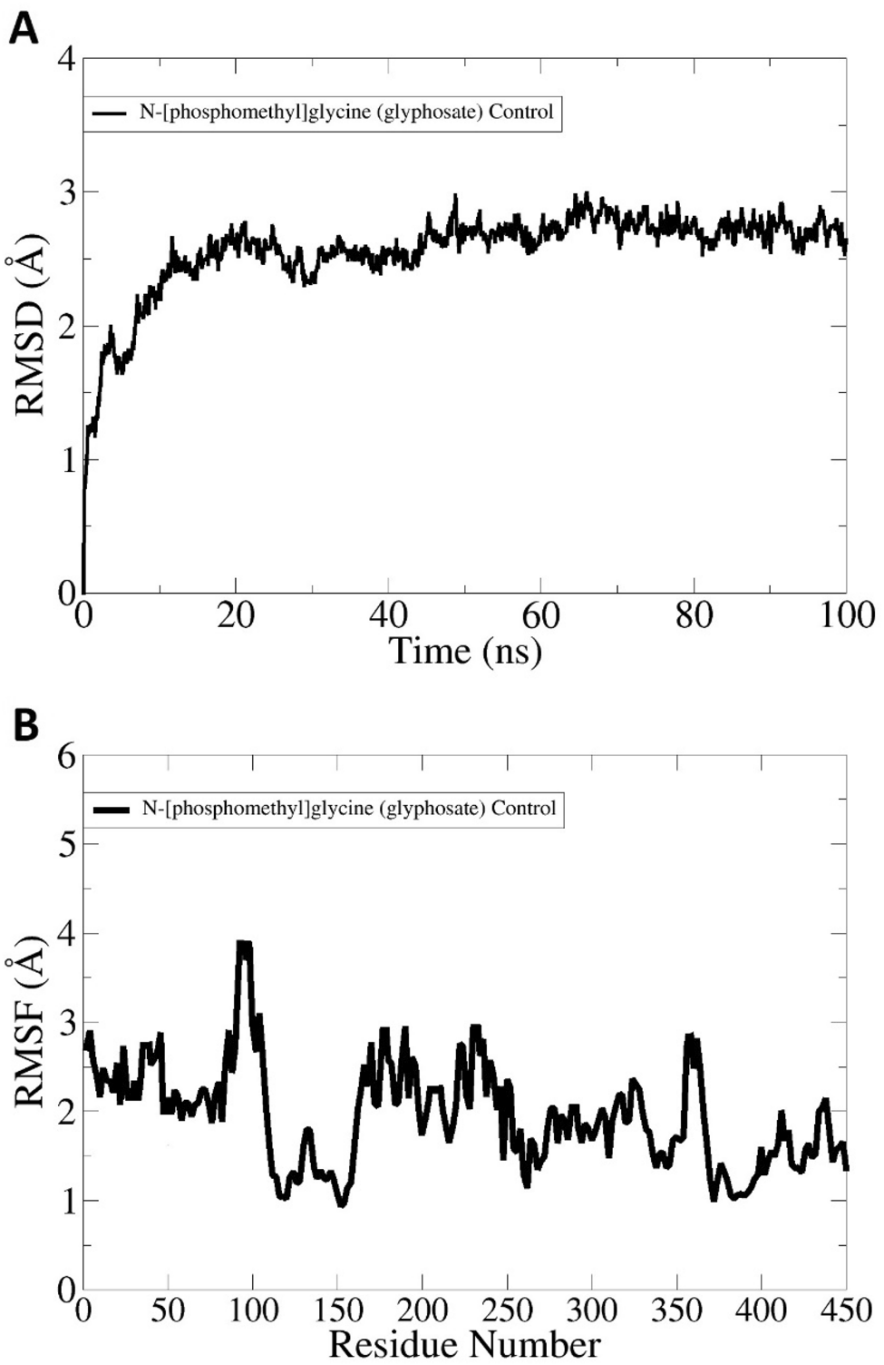
Molecular dynamics simulation of EPSP synthase in the presence of control. (**A)** RMSD, and (**B)** RMSF.

For comparative analysis, the N-[phosphomethyl]glycine (glyphosate) control was also used in molecular dynamics simulation. The RMSD of control was seen more stable as like Top-2 ad Top-3 despite of some initial deviations. The mean RMSD of control system is 2.4 Å wile mean RMSF is 2.3 Å. The EPSP synthase RMSD and RMSF in the presence of control is given I Fig. 6A, B, respectively.

### Inter molecular interactions RDF analysis

**Figure 7:**
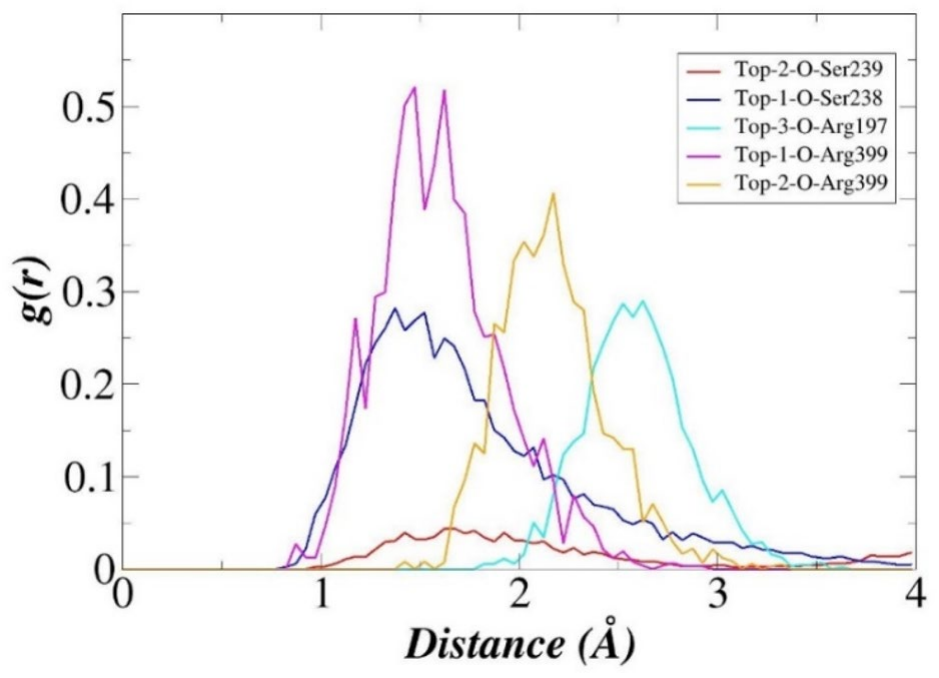
RDF plots of hydrogen bonds between the compounds and enzyme hotspot residues are substantial for engaging the compounds at the docked site.

RDF is a pair correlation function that estimates the density distribution of interacting radii significant in holding the ligand at the docked site^55,73^. RDF plots for key hydrogen bonding residues were generated to get an insight into the interatomic association of said interactions during simulation period (Fig. 7). For Top-1, two interactions engaging Ser238 and Arg399 residues were plotted that were revealed to be consistent in terms of density distribution. Two maximum density distribution points were observed for Top-1 oxygen atom and Arg399; first at 1.48 Å with *g(r)* of 0.51, followed by second distribution at 1.62 Å with *g(r)* of 0.50. For the second interaction of Top-1, Ser238 is attached to the compound with maximum interatomic distribution of 0.28 at a distance of 1.36 Å. It can be concluded from both interactions plot that said interactions are vital for keeping the ligand in close vicinity of the active pocket. Top-2 interaction with Ser239 played less contribution with variable g(r) values at different distances, though the interaction seems to play a critical role in compound recognition and binding. Interaction with Arg399 is more stable and density distribution is maximum at 2.16 Å with *g(r)* value of 0.40. Top-3 compound contact with Arg197 is more uniform at a distance of 2.6 with *g(r)* score of 0.28. All these plots suggest high affinity binding of the compounds to enzyme and formation of strong stable complexes. As interactions distance patterns between the compounds and enzyme active site residues are not much affected during simulation time, it can be inferred that binding mode of the compounds is not changed at the enzyme active pocket. As a result of which the interactions network of the compounds with respect to the enzyme remain consistent and in close distance based on which higher affinity of the compounds for the enzyme can be interpreted.

### Binding free energies estimation

To validate the affinity of compounds to EPSP, post simulation processing of MM-PB/GBSA was performed to yield different free energies of the complexes. MM-PBSA and MM-GBSA are considered reliable for this purpose as they are more accurate compared to docking predictions^27^. The MM-PBSA is computationally expensive than MM-GBSA but more reliable and accurate. Both methods were used to cross validate the findings. Table 1 tabulated the different binding free energies calculated by MM-PBSA and MM-GBSA methods. In MM/GBSA, the net binding free energy of the systems is −50.99 kcal/mol for Top-1, −49.5 kcal/mol for Top-2, and −46.54 kcal/mol for Top-3. In MM/PBSA, the total binding energy is −13.82 kcal/mol, −12.65 kcal/mol and −14.41 kcal/mol for Top-1, Top-2 and Top-3, respectively. In both methods, the net electrostatic energy, van der Waals and non-polar solvation energies have positive contributions to the net binding energy whereas the polar solvation energy is less favorable in complex formation. The control complex in contrast has a net MM-GBSA and MM-PBSA binding energy of 12.95 kcal/mol ad −5.75 kcal/mol, respectively. The entropy energy of Top-1, Top-2, Top-3 and control is 10.45 kcal/mol, 8 kcal/mol, 14 kcal/mol and 15 kcal/mol, respectively. The values indicate that entropy contribution is highly unfavorable to systems stability. However, as the net binding energy of the filtered compounds systems is very good, the entropy energy does not impact intermolecular binding significantly.

**Table 1:** MM-GBSA and MM-PBSA net binding energy of the compounds/control presented for each energy component.

Compound	ΔG binding	ΔG electrostatic	ΔG bind van der Waals	ΔG bind gas phase	ΔG polar solvation	ΔG non polar solvation	ΔG solvation
**MM-GBSA**							
Top-1	−24.15	−20.81	−30.18	−50.99	30.51	−3.67	26.83
Top-2	−28.44	−24.38	−24.87	−49.25	24.15	−3.33	20.81
Top-3	−22.44	−19.03	−27.51	−46.54	27.65	−3.54	24.10
Control	−12.95	−11.41	−26.02	−37.43	26.00	−1.52	24.48
**MM-PBSA**							
Top-1	−13.82	−20.81	−30.18	−50.99	50.68	−13.51	37.17
Top-2	−12.65	−24.38	−24.87	−49.25	44.06	−7.21	36.85
Top-3	−14.41	−19.03	−27.51	−46.54	50.71	−18.58	32.13
Control	−5.75	−10.00	−22.00	−32.00	27.25	−1.00	26.25

### Hotspot residues energy contribution

**Figure 8:**
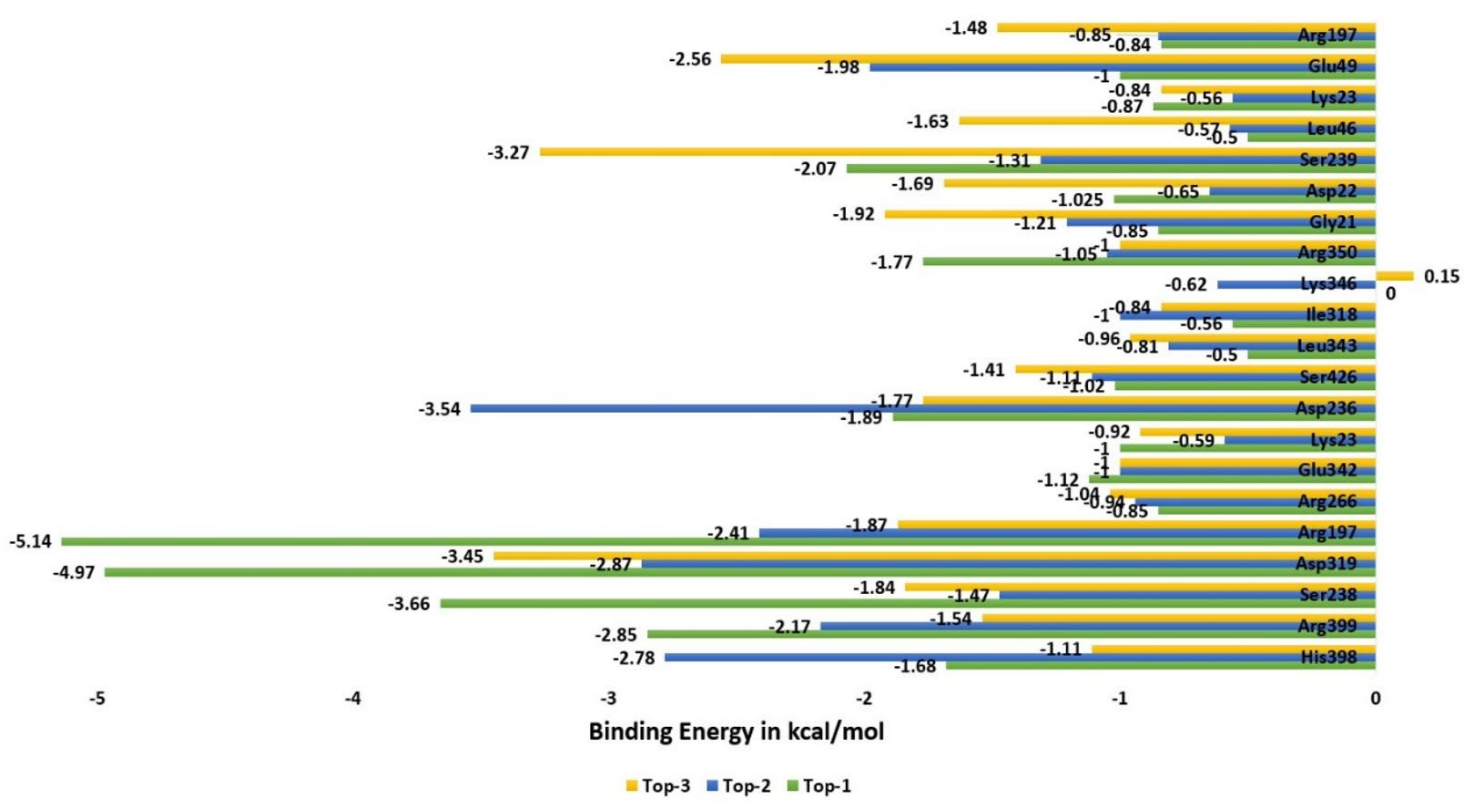
Net MM-GBSA binding free energy value of EPSP residues that are in direct contact with the compounds and formed the active pocket of enzyme.

Further investigation of the hotspot residues from the enzyme active pocket was done to understand their contribution to the total MM-GBSA binding energy. This was accomplished by decomposing the net energy into residues of the enzyme. Residues with the binding free energy of ≤ 1 kcal/mol were termed hotspot as they are significantly involved in interactions with the compounds. Residues like His398, Arg399, Ser238, Asp319, Arg197, Arg266, Glu342, Lys23, Asp236, Ser426, Leu343, Ile318, Lys346, Arg350, Gly21, Asp22, Ser239, Leu46, Lys23, Glu49, and Arg197 all contribute favourably to the binding of compounds. The binding energy of each of these residues is presented in Fig. 8.

### WaterSwap based binding free energy calculation

**Figure 9:**
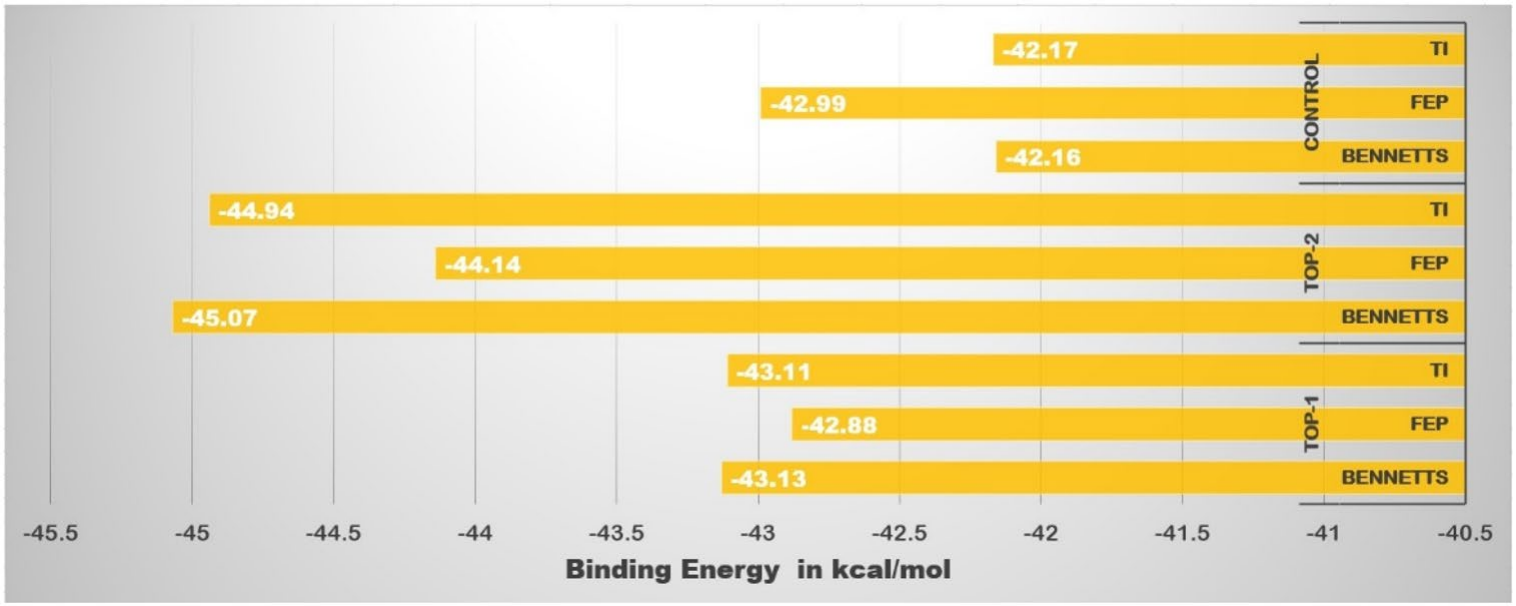
WaterSwap based absolute binding free energy of each compound estimated by TI, FEP, and BENNETTS methods.

The MM-PBSA and its counterpart MM-GBSA calculate the Gibbs free energy based on snapshot selected at regular intervals from simulation trajectories. As there is variation in the ligand conformation during simulation, it’s very difficult to predict which part of the ligand contributes significantly to the net binding energy^58^. Also, the use of an implicit model in these methods skips the role of water molecules in bridging the ligand and protein^59^. Such limitations can easily be overcome in WaterSwap. As can be seen in Fig. 9 the complexes binding energy is well converged (the difference in the binding energy value estimated by different methods is very small) and is quite stable (high intermolecular binding affinity) as predicted by all three WaterSwap binding energy methods.

### Alanine scanning analysis

The active residues of the enzyme that contribute highly to the net binding free energy and are involved in robust interaction with the inhibitor were mutated to decipher their functional significance. In specific, four residues: Arg197, Ser238, Ser239, and Arg399 were mutated to alanine to bring native structural changes in the enzyme but do not affect the overall conformation of the enzyme. By doing so, we observed a decline in the contribution of these residues as tabulated in Table 2.

**Table 2:** Mutated residues binding energy score in kcal/mol.

Residue	Top-1	Top-2	Top-3
Arg197	−2.40	−2.41	−1.87
Ser238	−1.91	−0.14	−1.11
Ser239	−0.71	−1.0	−3.1
Arg399	−0.65	−1.07	−0.95

### Druglikeness, medicinal chemistry, pharmacokinetics and toxicity analysis of the compounds

The loss of drugs owing to poor pharmacokinetics in drug development procedures leads to higher development expenses. Screening of promising drugs has been greatly improved by the availability of in silico pharmacokinetic tools^74^. Therefore, a detailed pharmacokinetic analysis of the top hit molecules was done to assist chemists to optimize the structure while maintaining an acceptable range of pharmacokinetics. Table 3 provides a detailed description of the pharmacokinetics of the screened compounds along with druglikeness, medicinal chemistry, and several toxicity analysis. According to the rules of medicinal chemistry, drug absorption is of prime importance and should be assessed in the in silico pharmacokinetics studies first^75^. The screened molecules are observed as water soluble and indicate good oral bioavailability as predicted by methods such as estimating aqueous solubility (ESOL), Ali’s method and SILICOS-IT methods of SwissADME^63^. The molecules also have high gastrointestinal absorption thus ensuring high concentration of the drugs can be reached to the target site. The molecules have good skin penetrable potential making them suitable for transdermal delivery. The molecules ADME properties are within the scope of known druglike rules including the prominent Lipinski rule of five^76^, Ghose^77^, Veber^78^, Egan^79^, and Muegge^80^ rules. This implies that the drugs are suitable candidates to show favorable pharmacokinetics and might have good oral bioavailability. The analysis of medicinal chemistry confirmed the molecules to have good synthetic accessibility and granted zero alerts for pans assay interference structures (PAINS)^43^. The zero alert for PAINS demonstrates the compounds to selectively bind to the EPSP. Screened molecules are predicted to have poor permeability for blood brain barrier (BBB)^81^ and are unable to cross the central nervous system. The molecules are non-inhibitors of cytochrome P450 allowing functional oxidation of xenebiotics^82^. The molecules are predicted to show no hepatotoxicity, skin sensitization, AMES, and carcino toxicity, all these parameters suggesting the molecules to be potential candidates subject to further experimental exploration.

**Table 3:** Predicted druglikeness and ADMET analysis of the compounds.

Property	Compound		
Physicochemical properties	Top-1	Top-2	Top-3
Formula	C17H31NO6S	C12H14O3	C14H18O4
Molecular weight	377.50 g/mo	206.24 g/mol	250.29 g/mol
Num. heavy atoms	25	15	18
Num. arom. heavy atoms	0	6	6
Fraction Csp3	0.88	0.33	0.43
Num. rotatable bonds	11	1	5
Num. H-bond acceptors	6	3	4
Num. H-bond donors	3	2	3
Molar refractivity	101.54	58.08	68.50
TPSA	132.60 Å^2^	49.69 Å^2^	77.76 Å^2^
**Lipophilicity**			
Consensus Log Po/w	1.87	1.44	1.43
Water solubility	Soluble	Soluble	Soluble
**Pharmacokinetics**			
GI absorption	High	High	High
BBB permeant	No	Yes	No
P-gp substrate	Yes	No	No
CYP1A2 inhibitor	No	No	Yes
CYP2C19 inhibitor	No	No	No
CYP2C9 inhibitor	No	No	No
CYP2D6 inhibitor	No	No	No
CYP3A4 inhibitor	No	No	No
Log Kp (skin permeation)	−6.96 cm/s	−6.59 cm/s	−6.35 cm/s
**Druglikeness**			
Lipinski	Yes	Yes	Yes
Ghose	Yes	Yes	No; 1 violation: WLOGP < −0.4
Veber	No; 1 violation: Rotors > 10	Yes	Yes
Egan	No; 1 violation: TPSA > 131.6	Yes	Yes
Muegge	Yes	Yes	Yes
Bioavailability Score	0.56	0.55	0.55
**Medicinal chemistry**			
PAINS	0 alert	0 alert	0 alert
Brenk	0 alert	0 alert	0 alert
Synthetic accessibility	4.72	3.93	3.72
**Toxicity**			
Hepatotoxicity	Yes	No	No
Skin sensitisation	No	No	No
*T.pyriformis* toxicity	0.285 log ug/L	0.208 log ug/L	0.232 log ug/L
AMES toxicity	No	No	No
Minnow toxicity	1.926 log mM	1.829 log mM	1.647 log mM
Carcino mouse	No	No	No
**Excretion**			
Total clearance	0.608 log ml/min/kg	0.236 log ml/min/kg	0.627 log ml/min/kg
Renal OCT2 substrate	No	No	No

## Conclusions

Compounds of natural origin and their molecular frameworks play a significant contribution in the discovery of new drugs^40^. This can be evidenced by the approval of two-thirds of natural source small molecule drugs from January 1981 to September 2019^83^. In particular, natural compounds from oceans have immense potential to become good drug molecules because of extreme biodiversity marine organism secondary metabolites^84^. Therefore, marine compounds are now considered a hotspot in recent drug research and development. Considering the importance of vast therapeutic potentials of marine drugs, herein, we used the CMNPD database to identify potent inhibitory molecules against WHO's top priority list *A. baumannii* bacterial pathogen. Three molecules CMNPD31561, CMNPD28986, and CMNPD28985 as Top-1, Top-2, and Top-3 were virtually scored as potential blockers of the EPSP synthase, thus might stop the growth and survival of *A. baumannii*. These findings were validated by several computational analysis including MD simulation, MM-GB/PBSA, and WaterSwap binding energies that certain that the compounds interact with the enzyme with good affinity and formed strong intermolecular interactions. The interactions between the enzyme and compounds are uniform and are present in high density throughout the length of simulation time. From future perspective, the compounds are good drug candidates as they clear all prominent druglike rules and have sound pharmacokinetics. In a nutshell, the compounds are concluded to show good biological potency for EPSP synthase enzyme and thus required in vivo and in vitro experimental testing.

## Supplementary Information

Supplementary Information.

## Data Availability

All the data is available within the manuscript and supplementary material.
