# Supplementary material for: Integrated computer-aided drug design and biophysical simulation approaches to determine natural anti-bacterial compounds for Acinetobacter baumannii
Source: Sci Rep. 2022 Apr 21;12:6590. doi: 10.1038/s41598-022-10364-z (PMC9023527; doi:10.1038/s41598-022-10364-z)
Supplement: Supplementary file 1 — Supplementary Information. [file 41598_2022_10364_MOESM1_ESM.docx]

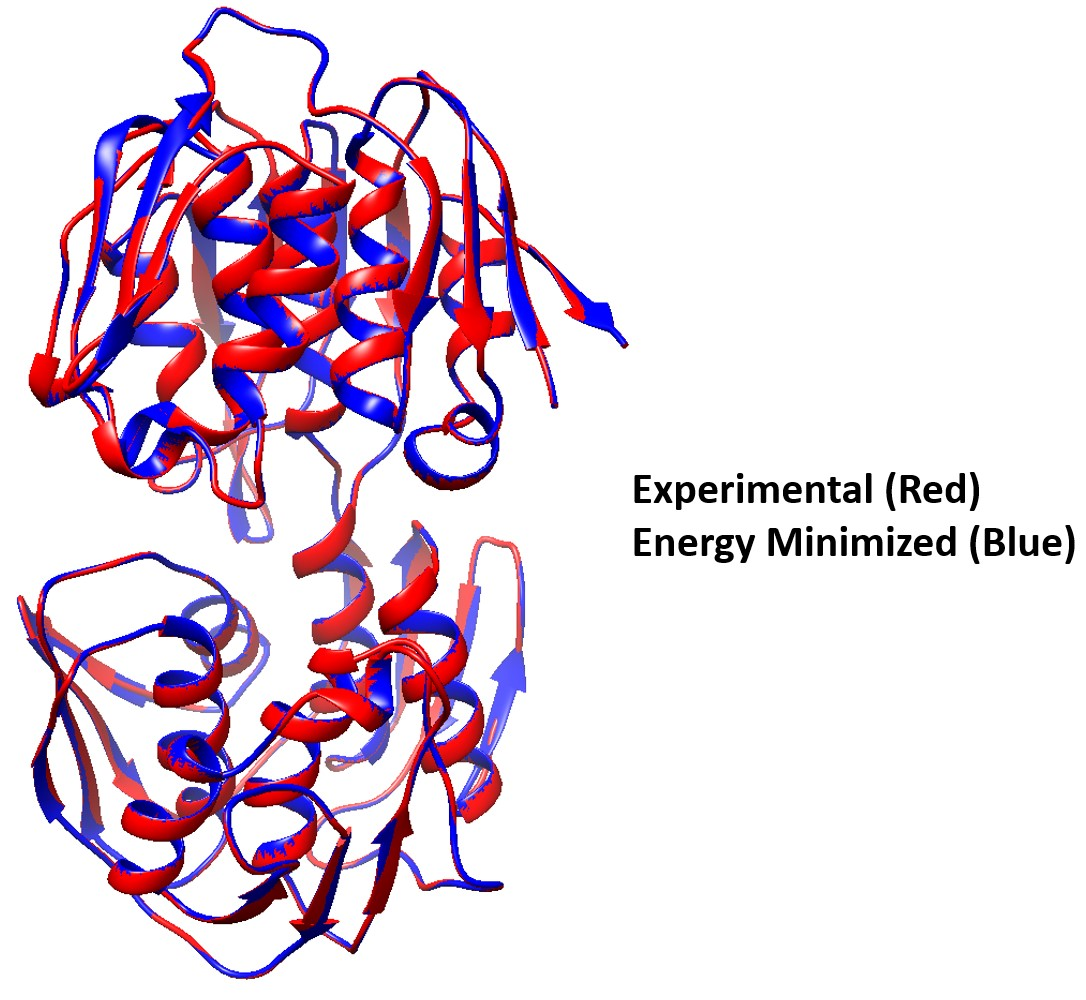


**Fig. S1.** The superimposed energy minimized EPSP enzyme over experimental EPSP enzyme.


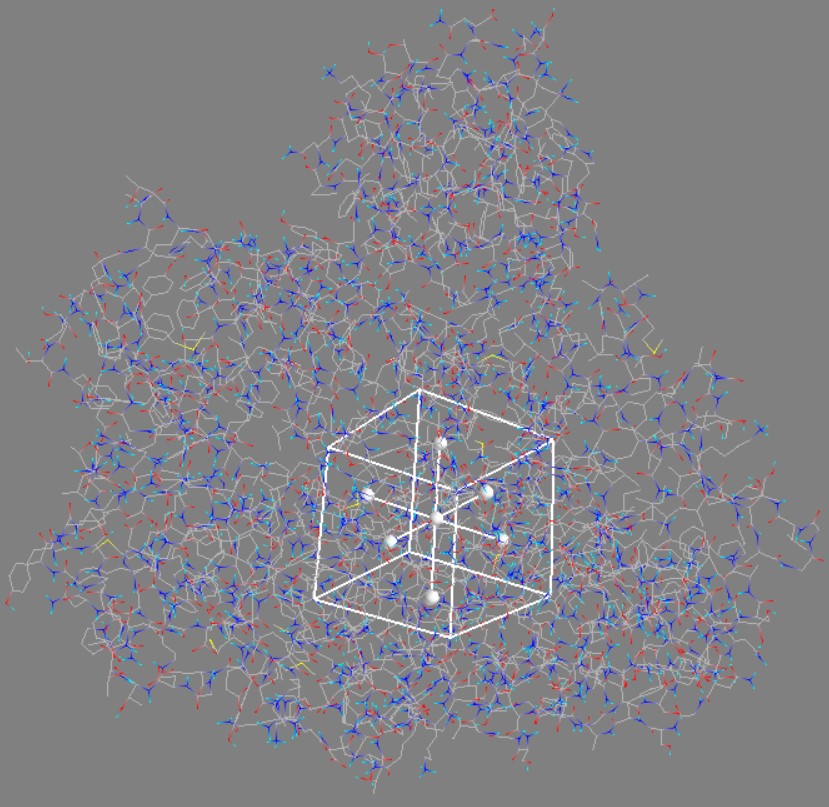


**Fig. S2.** The presentation of target protein with dock box.
